# Safety of endoscopy in cancer patients on antiangiogenic agents: A retrospective multicenter outcomes study

**DOI:** 10.1371/journal.pone.0176899

**Published:** 2017-05-04

**Authors:** Toufic Kachaamy, Digant Gupta, Persis Edwin, Pankaj Vashi

**Affiliations:** Cancer Treatment Centers of America, 5900 Broken Sound Parkway, Boca Raton, Florida, United States of America; Virginia Mason Medical Center, UNITED STATES

## Abstract

**Background/Aims:**

The use of antiangiogenic agents (AAs) in cancer treatment has increased because they offer survival benefit in combination with cytotoxic chemotherapy. Given their potential to cause gastrointestinal (GI) perforation and bleeding, it is currently recommended that AAs be held for 28 days before and after surgery. However, there are no specific guidelines which address their use around endoscopic procedures because data regarding the safety of endoscopy in cancer patients while on AAs is scarce despite the fact that these patients often require endoscopy. This study investigated the safety of endoscopy in cancer patients receiving AAs.

**Methods:**

This is a retrospective multicenter study of a consecutive case series of 445 cancer patients undergoing endoscopy within 31 days of administration of AAs at 5 specialized cancer centers between April 2008 and August 2014. Endoscopies were classified into two different categories based on the risk of GI bleeding and perforation: low and high. The primary outcome measures were procedure-related adverse events (AEs) and death within 30 days of endoscopy. The severity of AEs was classified according to the common terminology criteria for adverse events (CTCAE) version 4.0. The incidence of AEs and mortality was calculated using the total number of patients as the denominator.

**Results:**

445 cancer patients with a mean age of 54 years underwent a total of 545 endoscopies. Median time duration from AAs to endoscopy was 11 days. Of 545 endoscopic procedures, 398 (73%) were low-risk and 147 (27%) were high-risk. There were 3 procedure-related AEs: esophageal perforation (grade 3) two days after an EGD, pancreatitis (grade 5) a day after failed ERCP, and bleeding from the gastrostomy site (grade 1) two days after an EGD. Of 445 patients, 29 (6.5%) died within 30 days of the procedure with no deaths deemed procedure-related. The most common causes of death were terminal cancer (n = 10), hepatic decompensation (n = 5) and sepsis (n = 4).

**Conclusion:**

In this retrospective study, the rate of endoscopy-related AEs in patients on AAs appears to be low when performed in specialized cancer centers. However, future prospective studies are needed to confirm this finding.

## Introduction

Antiangiogenic agents (AAs) such as bevacizumab and aflibercept offer survival benefits in combination with cytotoxic chemotherapy in many metastatic cancers including colon, renal, non–small cell lung and breast [1–6]. These agents are inhibitors of vascular endothelial growth factor (VEGF), a glycoprotein that is overexpressed in many solid tumors and is a key regulator of the angiogenesis process [7;8]. It plays a very important role in the regulation of tumor-related angiogenesis, which in turn is crucial for tumor growth, invasion and metastasis [9]. Higher VEGF expression is associated with greater tumor invasiveness and metastatic ability. In addition, VEGF inhibits endothelial cell apoptosis and increases tumor interstitial pressure, reducing the penetration of cytotoxic drugs into the tumor mass [10].

The use of AAs has expanded significantly in the last few years leading to an increased awareness of potential toxicities associated with them. The mechanism of action described above is also the basis of toxicities associated with the use of anti-VEGF agents [9]. The toxicity profile of anti-VEGF agents includes hypertension, proteinuria, bleeding [including gastrointestinal (GI) bleeding], GI perforation, impaired wound healing, and arterial/venous thromboembolism [8;11–13]. The incidence and the severity of these toxicities varies greatly across studies, however, bleeding stands out as the most severe and difficult to manage [9;10]. Among the anti-VEGF agents, bevacizumab retains the highest frequency of bleeding including epistaxis, hemoptysis, hematemesis, GI or vaginal bleeding, and brain hemorrhage [8;14].

Some retrospective studies in the literature have demonstrated a higher rate of post-operative adverse events (AEs) with bevacizumab as compared to the control group, although the findings failed to reach statistical significance in all these studies possibly because of small sample sizes [7;15;16]. However, a meta-analysis of 22 randomized controlled trials found that addition of bevacizumab to cancer chemotherapy significantly increased the risk of high-grade bleeding (relative risk 1.60, 95% CI 1.19–2.15). The risk of high-grade bleeding was dose-dependent with relative risks of 1.27 (95% CI 0.95–1.71) and 3.02 (95% CI 1.85–4.95) among patients receiving bevacizumab at 2.5 and 5 mg/kg per week respectively. The overall incidence of high-grade bleeding among patients receiving bevacizumab was 2.8% (95% CI 2.1–3.8) [9]. Similarly, a more recent meta-analysis of 34 randomized controlled trials found that patients receiving bevacizumab in combination with taxanes and/or platinum agents had a significantly increased risk of fatal AEs with a relative risk of 1.29 (95% CI:1.05–1.57) compared to patients receiving chemotherapy alone. Of the reported causes of fatal AEs, the rates of hemorrhage, pulmonary embolism, neutropenia, GI tract perforation, and cerebrovascular accident were higher on the bevacizumab treatment arms [17].

In light of the literature described above, it is recommended that elective invasive surgery be delayed for over 40 days after the completion of bevacizumab therapy, the half-life of which is 20 days, to adequately prevent toxicities resulting from inhibition of VEGF-mediated physiological processes including wound healing [8]. In addition, it has been recommended that postoperative re-initiation of bevacizumab in surgical patients should wait at least 28 days and the surgical incision should be fully healed to prevent an increased risk of wound healing complications [7;15;18–20].

Similar to the surgical literature described above, some studies have demonstrated that endoscopic interventions such as colonic stenting are associated with a higher GI perforation rate in patients on AAs [21–23]. However, the evidence base for this association is weak and causality has not been conclusively established. Moreover, unlike major surgical procedures, the endoscopic procedures typically lead to injuries limited to the mucosa or superficial submucosa and the process of healing might not require new vascular formation. As a result, recommendations regarding the use and timing of AAs in surgery cannot be extrapolated to endoscopic procedures until more definitive evidence becomes available on the safety of endoscopy in patients on AAs. This study investigated the incidence of AEs in patients undergoing endoscopy within 31 days of administration of AAs.

## Methods

### Study design and patient population

This is a retrospective multicenter study of a consecutive case series of cancer patients treated at five Cancer Treatment Centers of America^®^ (CTCA) hospitals specializing in cancer care in the United States of America. The inclusion criteria for this study were: 1) patients who underwent an endoscopic procedure between April 2008 and August 2014 at all 5 CTCA hospitals and 2) patients who were administered AAs within 31 days prior to the date of endoscopy. There were no exclusion criteria for this study, and all consecutive patients who underwent endoscopy within 31 days of receiving AAs were included in the analysis.

Patients who underwent an endoscopic procedure while on AAs were identified through the electronic medical records. Timing of the administration of AAs and endoscopy was obtained. In accordance with the previously published literature[24], endoscopies were classified into two different risk categories: “low” risk when an endoscopy was performed for diagnostic purposes only (including mucosal biopsies) and “high” risk when therapeutic maneuvers were performed including endoscopic retrograde cholangiopancreatograpy (ERCP), gastrostomy tube placement, hemostasis, endoscopic celiac neurolysis, structure dilation, endoscopic ultrasound (EUS) fine needle aspiration and snare polypectomy. This classification was used because the risk of GI bleeding and perforation is higher with therapeutic maneuvers and lower with diagnostic procedures. For example, an endoscopy with therapeutic maneuver such as ERCP with sphincterotomy or esophageal dilation involves a large breach of the mucosal and submucosal tissue with the highest risk of bleeding or perforation either due to the maneuver itself or due to lack of healing while on AAs.

The present study was conducted according to the guidelines laid down in the Declaration of Helsinki and was approved by the CTCA Institutional Review Board. The need for informed consent was waived by the Institutional Review Board because the analysis used anonymous clinical data that were obtained after each patient agreed to treatment by written consent. This study involved collection of existing data from patient records in such a manner that subjects cannot be identified, directly or through identifiers linked to the subjects. Patient records/information was anonymized and de-identified prior to analysis.

### Statistical analysis

Data are presented as frequencies and percentages. The primary outcome measures were procedure-related AEs and deaths within 30 days of endoscopy. AEs were classified according to the common terminology criteria for adverse events (CTCAE) version 4.0 which defines an AE as any unfavorable and unintended outcome associated with the use of a medical treatment or procedure that may or may not be considered related to the medical treatment or procedure. The severity of AEs was measured using a grade of 1 through 5 (1 = mild; 2 = moderate; 3 = severe; 4 = life-threatening; 5 = death). The incidence of AEs and mortality was calculated using the total number of patients as the denominator. All data were analyzed using IBM SPSS version 23.0 (IBM, Armonk, NY, USA).

## Results

### Patient characteristics

The final study population consisted of 445 consecutive patients from five hospitals specializing in cancer care. The mean age of patients at the time of the endoscopic procedure was 54 years (standard deviation, 10.3 years; range 21 to 82 years). The most common cancer types were colorectal, lung and breast, and the majority of patients had stage III or IV disease at the time of diagnosis. **Table 1**describes the patients’ baseline characteristics.

**Table 1:** Baseline patient characteristics (N = 445).

Characteristic	Categories	Number (Percent)
Gender	Male	198 (44.5)
	Female	247 (55.5)
Cancer Site	Colorectal	193 (43.4)
	Lung	60 (13.5)
	Breast	44 (9.9)
	Ovarian	20 (4.5)
	Pancreas	16 (3.6)
	Others	105 (23.6)
Cancer Stage at Diagnosis	Stage I	29 (6.5)
	Stage II	55 (12.4)
	Stage III	106 (23.8)
	Stage IV	184 (41.3)
	Undetermined	71 (16)
CTCA Hospital	Southwestern	220 (49.4)
	Eastern	86 (19.3)
	Midwestern	64 (14.4)
	Western	56 (12.6)
	Southeastern	19 (4.3)

(CTCA = Cancer Treatment Centers of America)

### Endoscopic procedures

There were 445 patients who underwent a total of 545 endoscopies. The median time duration between the use of AAs and the endoscopic procedure was 11 days. **Table 2**displays the types of endoscopic procedures along with their risk classification. Of a total of 545 endoscopic procedures, 398 (73%) were low-risk and 147 (27%) were high-risk. The most common procedure was esophagogastroduodenoscopy (EGD) followed by colonoscopy, EUS, sigmoidoscopy and ERCP. Of a total of 125 colonoscopies, 124 were low-risk (diagnostic only) whereas 1 was considered high-risk due to the use of the hemostasis maneuver. Similarly, of a total of 24 sigmoidoscopies, 23 were low-risk (diagnostic only) whereas 1 was considered high-risk due to the use of the snare polypectomy maneuver. In the 12 patients who underwent ERCP, the following maneuvers were performed: cannulation (n = 1), dilation and stent change (n = 1), sphincterotomy and stent placement (n = 6), sphincterotomy, dilation and stent placement (n = 2), sphincterotomy and brushings (n = 1) and stent change only (n = 1).

**Table 2:** Risk Classification of endoscopic procedures (N = 545).

Endoscopic Procedure	Low-risk	High-risk	Total
EGD	235	96	331
Colonoscopy	124	1	125
EUS	16	37	53
Sigmoidoscopy	23	1	24
ERCP	0	12	12
**Total**	398	147	**545**

(EGD = esophagogastroduodenoscopy, ERCP = endoscopic retrograde cholangiopancreatograpy, EUS = endoscopic ultrasound)

### Adverse events and mortality

Among 445 patients, 3 procedure-related AEs occurred (0.7%) within 30 days of the procedure, as shown in **Table 3**. One patient who underwent gastrostomy tube placement experienced bleeding (grade 1) from the gastrostomy site. This resolved without the need for transfusion or hospitalization. Another patient who underwent an upper endoscopy for dysphagia presented the following day with fever and what appeared to be esophageal perforation (grade 3) on imaging. The patient was hospitalized, treated with intravenous antibiotics and an esophagogram showed no evidence of contrast extravasation. The patient’s symptoms resolved with conservative management and surgical intervention was not needed. The third patient had an upper endoscopy with attempt at ERCP for cholangitis. Pancreatic cannulation was performed but biliary cannulation failed. The patient developed pancreatitis (grade 5) but it was decided that no further interventions will be attempted because of patient’s advanced disease. The patient died from underlying sepsis. The time interval between the last dose of AAs and the endoscopy procedure in these 3 patients was 21, 20 and 18 days respectively.

**Table 3:** Procedure-related adverse events (N = 445).

AE	AE Grade	Case Description	Indication for GI Procedure	GI Procedure	GI Procedure Risk	Time between AAs and GI Procedure (Days)	AE Timing (Days Since GI Procedure)	AE Management
Esophageal perforation	3	Female, 66 years, cholangiocarcinoma stage IV	Dysphagia	EGD with biopsy only	Low	20	2	IV antibiotics with resolution
Pancreatitis	5	Female, 49 years, colorectal cancer stage IV	Jaundice and cholangitis	EGD/ERCP aborted after failed cannulation	Low	18	1	ERCP failed and patient died from underlying sepsis
Bleeding from the gastrostomy site	1	Female, 58 years, non-small cell lung cancer stage IV	Malnutrition	EGD for PEG tube placement	High	21	2	No intervention needed

(AAs = antiangiogenic agents, AE = adverse events, EGD = esophagogastroduodenoscopy, ERCP = endoscopic retrograde cholangiopancreatograpy, EUS = endoscopic ultrasound, GI = gastrointestinal, IV = intravenous, PEG = percutaneous endoscopic gastrostomy)

Of a total of 445 patients, 29 died within 30 days of the procedure. All 29 patients had stage IV disease at diagnosis. The causes of death (based on chart reviews and death certificates) are reported in **Table 4**. The most common causes of death were terminal cancer (n = 10), hepatic decompensation secondary to extensive tumor burden (n = 5) and sepsis (n = 4). In 4 patients, the cause of death was not available, however, a detailed review of the records of these patients revealed that none of the deaths were related to the endoscopic procedures. None of the 29 patients who died within 30 days of the procedure had a procedure-related GI bleeding or perforation. One patient who presented with cholangitis (#15 in Table 4) developed pancreatitis a day after an attempt at ERCP and biliary drainage failed. This patient died of sepsis within 30 days of the procedure. This patient is also included in Table 3 describing AEs.

**Table 4:** Causes of death among patients who died within 30 days of endoscopy (N = 445).

No.	GI Procedure	Indication for GI Procedure	Cancer Site and Stage	Cause of Death
1	Colonoscopy	Rectal bleeding	Colon, IV	Hepatic decompensation
2	Colonoscopy	Suspected colonic obstruction	Colon, IV	Hepatic decompensation
3	Colonoscopy	Unexplained diarrhea	Colon, IV	Cancer
4	EGD	Odynophagia	Unknown primary, IV	Brain metastasis and sudden loss of consciousness
5	EGD	Vomiting	Breast, IV	*Records unavailable*
6	EGD	Malnutrition	Breast, IV	Cancer
7	EGD	Dysphagia	Breast, IV	Respiratory failure from malignant pleural effusion
8	ERCP	Jaundice	Breast, IV	*Records unavailable*
9	EGD	Rectal bleeding	Breast, IV	Febrile neutropenia and sepsis
10	EGD	Malnutrition	Breast, IV	Cancer
11	EGD	GI bleeding	Breast, IV	Cirrhosis, breast cancer and multi-organ failure
12	EGD	GI bleeding	Colon, IV	*Records unavailable*
13	EGD	Chronic small bowel obstruction	Colon, IV	Cancer
14	EGD	Malnutrition	Colon, IV	Cancer
15	EGD/ERCP aborted after failed cannulation	Jaundice and Cholangitis	Colon, IV	Underlying sepsis
16	EGD	Dysphagia	Colon, IV	Aspiration pneumonia
17	EGD	G-tube dysfunction	Colon, IV	Cancer
18	EGD	Malnutrition	Endometrial, IV	Cancer
19	EGD/ERCP not attempted because of severe esophageal stenosis	Jaundice	Esophageal, IV	Cancer
20	EGD	Esophageal stent migration	Esophageal, IV	Cancer
21	EGD	dysphagia	Lung, IV	Cancer
22	EGD	Malnutrition	Lung, IV	Respiratory failure from malignant pleural effusion
23	ERCP	Jaundice and sepsis	Breast, IV	Sepsis
24	ERCP	Jaundice and cholangitis	Colon, IV	Sepsis
25	EUS	Abdominal pain	Cholangiocarcinoma, IV	Hepatic decompensation
26	EUS	Abdominal pain	Colon, IV	Hepatic decompensation
27	EUS	Abdominal pain	Ovarian, IV	Hepatic decompensation
28	EUS	Abdominal pain	Pancreatic neuroendocrine, IV	Pneumonia
29	Sigmoidoscopy	Suspected colonic obstruction	Colon, IV	*Records unavailable*

(EGD = esophagogastroduodenoscopy, ERCP = endoscopic retrograde cholangiopancreatograpy, EUS = endoscopic ultrasound, GI = gastrointestinal)

## Discussion

No data exists on the safety of endoscopy in patients on AAs. In this study, we investigated the incidence of AEs and mortality in cancer patients undergoing endoscopy within 31 days of administration of AAs.

The key finding from this study is that endoscopy (whether diagnostic or therapeutic) is well tolerated in patients on AAs given the low AE incidence rate of 0.7% (3/445). Even though the 30-day “overall” mortality in our study was high at 6.5% (29/445), none of the deaths were related to the endoscopic procedure. This high mortality can be attributed to the fact that over 60% of our patients had advanced stage cancer (stages III and IV). In 4 patients where the cause of death was not available, a detailed review of their charts indicated a poor likelihood of their deaths being procedure-related. Even if we assumed these 4 patients died from procedure-related perforation or severe GI bleeding, the rate of AEs in this study would become 1.6% (7/445) and the rate of 30-day “procedure-related mortality” would become 0.9% (4/445). This presumed AE rate of 1.6% is in line with the reported rate of spontaneous perforation or bleeding associated with the use of AAs in the absence of endoscopy [9;17]. This suggests that while endoscopic procedures performed on patients receiving AAs do carry a risk of AEs, this risk might be inherent to the advanced disease that these patients have.

Based on the above findings, we present here some practical recommendations for performing endoscopies in cancer patients receiving AAs. If endoscopy is elective, it should be scheduled around 2 days before the AAs are planned to be dosed. This time period represents the trough level of AAs in the patient’s system. If the endoscopy ends up being purely diagnostic with only mucosal biopsies and no high-risk maneuvers, our data suggests that these patients can continue with AAs as scheduled. If a high-risk maneuver is performed where AAs may increase the rate of AEs for the subsequent one to two weeks, a discussion should be held with the oncologist regarding temporarily holding the AAs for one to two weeks to allow complete healing. We would like to emphasize that discussions between the gastroenterologist and the oncologist should ideally occur before the endoscopy in order to adjust the threshold for maneuver performed based on the willingness of the oncologist to hold AAs. For emergency situations, given that the risk of endoscopy even with a therapeutic maneuver is low, we recommend proceeding with endoscopy regardless of the use of AAs. The informed consent process should however include a discussion about the fact that AAs are themselves associated with an increased risk of GI bleeding and perforation and these AEs can occur within thirty days after the endoscopic procedure.

There are several strengths of this study. First, the analysis included a consecutive case series of all eligible patients during a specific time period, thereby minimizing the possibility of selection bias. Second, the use of a heterogeneous cancer population from multiple cancer hospitals improves the generalizability of the study’s findings. Third, the use of mortality (a hard endpoint) as one of the primary outcomes provides data (currently unavailable in the literature) on severe AEs after endoscopy while on AAs. Our data can serve as the basis for a prospective study on the safety of endoscopy while on AAs, a study that is highly needed given the increasing role AAs are playing in cancer management and the important role that endoscopy plays in the diagnosis and treatment of cancer patients.

Some limitations of this study require careful acknowledgment. First, given the low incidence of complications, this study is under-powered to evaluate the factors associated with higher or lower risk of experiencing an endoscopy-related AE. As an example, we could not ascertain whether patients undergoing high-risk endoscopy procedures within a week of receiving AAs were more likely to experience an AE compared to patients undergoing a low-risk endoscopy procedure more than a week after receiving AAs. Second, this is a retrospective study using data not primarily collected for the purposes of research. It is possible that an emergency room visit or hospitalization outside the 5 hospitals in this study were undocumented and thus not reported or captured in the analysis. Similarly, patients might not have reported some AEs such as minor bleeding leading to an underestimation of the overall AE rate. Finally, the lack of a control group prevents us from investigating the question whether the risk of endoscopy-related AEs is higher in cancer patients receiving AAs compared to those not receiving them. Similarly, our study could not evaluate whether the morbidity and mortality is higher in cancer patients receiving both endoscopy and AAs compared to those receiving only AAs. However, as discussed above, a comparison of the AE rate in our study (where patients received both endoscopy and AAs) was found to be consistent with the AE rate reported in the literature (in patients receiving AAs in the absence of endoscopy). Prospective studies with large sample sizes and a control group (either cancer patients receiving AAs but not undergoing endoscopy or cancer patients undergoing endoscopy but not receiving AAs) are clearly needed to be able to conclusively determine the safety of endoscopy in patients receiving AAs. To that effect, our study can provide useful benchmark data for comparison.

## Conclusion

In this retrospective study, the rate of endoscopy-related AEs in patients on AAs appears to be low when performed in specialized cancer centers. However, future prospective studies are needed to confirm this finding.
